# Isolation and Identification of Endophytic Fungi from *Actinidia macrosperma* and Investigation of Their Bioactivities

**DOI:** 10.1155/2012/382742

**Published:** 2011-11-24

**Authors:** Yin Lu, Chuan Chen, Hong Chen, Jianfen Zhang, Weiqin Chen

**Affiliations:** ^1^College of Biological and Environmental Engineering, Zhejiang Shuren University, Hangzhou 310015, China; ^2^Hangzhou Botanical Garden, Hangzhou 310013, China

## Abstract

Endophytic fungi from the Chinese medicinal plant *Actinidia macrosperma* were isolated and identified for the first time. This was the first study to evaluate their cytotoxic and antitumour activities against brine shrimp and five types of tumour cells, respectively. In total, 17 fungal isolates were obtained. Five different taxa were represented by 11 isolates, and six isolates were grouped into the species of Ascomycete *Incertae sedis* with limited morphological and molecular data. Cytotoxic activity has been found in most isolates except AM05, AM06, and AM10. The isolates AM07 (4.86 **μ**g/mL), AM11 (7.71 **μ**g/mL), and AM17 (14.88 **μ**g/mL) exhibited significant toxicity against brine shrimp. The results of the MTT assay to assess antitumour activity revealed that 82.4% of isolate fermentation broths displayed growth inhibition (50% inhibitory concentration IC_50_< 100 **μ**g/mL). Moreover, AM07, AM11, and AM17 showed strong antitumour activity in all the cell lines examined. These results suggest that endophytic fungi in *A. macrosperma* are valuable for the isolation and identification of novel cytotoxic and antitumour bioactive agents.

## 1. Introduction

Endophytic fungi are mitosporic and meiosporic ascomycetes that asymptomatically reside in the internal tissues of plants beneath the epidermal cell layer, where fungi colonise healthy and living tissue via quiescent infections [1]. Their biological diversity is enormous, especially in temperate and tropical rainforests. The fungi are hosted in nearly 300,000 land plant species, with each plant hosting one or more of these fungi. Endophytic strains have been isolated from many different plants including trees (pine and yew), fodders (alfalfa, sorghum and clover), vegetables (carrot, radish, tomatoes, sweet potatoes, lettuce, and soybean), fruits (banana, pineapple, and citrus), cereal grains (maize, rice, and wheat), and other crops (sugarcane, marigold, and coffee) [2]. Moreover, endophytes comprise a rich and reliable source of genetic diversity and biological novelty and have been applied in pharmacology (e.g., the anticancer drug taxol) and agriculture [3].

*A. macrosperma* is a naturally wild kiwifruit endemic to China. As a traditional medicine, this plant is commonly known as “Cat Ginseng” because the volatile chemicals that are released from the aerial parts of the plant are sensitive to cats. The fresh leaves or twigs, which are used to heal wounds, are favoured by cats [4]. Moreover, *A. macrosperma* is reputed to counteract various ailments, including leprosy, abscesses, rheumatism, arthritis inflammation, jaundice, and abnormal leucorrhoea [5]. Previous studies have reported that the root and, stem of this plant are effective for treating cancers, especially lung cancer and cancer of digestive system [6]. Nevertheless, little is known about the chemical and biological activity of *A. macrosperma*.

Due to increasing consumption, the wild *A. macrosperma* has rapidly decreased and is exhausted in some areas based on our field and market investigations as well as folk inquiries. Much attention should be paid to the effective protection and sustainable development of this species. In recent years, we have, therefore, conducted a series of research projects focusing on the chemistry and tissue culture of *A. macrosperma* [7–10]. This study is a followup to our previous review and is a first step to examine whether the internal tissues of *A. macrosperma* are colonised by endophytic fungi. In addition, we isolated and identified each endophyte and investigated their biological activities.

## 2. Materials and Methods

### 2.1. Collection of Plant Material

Plant material was collected from fully matured *A. macrosperma* between August 2009 and November 2009 in a hilly region of Fuyang County in the Zhejiang Province in China. An identified specimen was housed in the Zhejiang University Herbarium (ZJUH) in China (Voucher number: HZU-A2009086). After plant selection, disease-free parts of the plant, that is, stem, root, and leaves, were excised with a sterile scalpel and placed in sterile plastic bags for storage at 4°C until use.

### 2.2. Isolation of Endophytic Fungi

The endophytes were isolated using a modified method described by Arnold et al. [11]. The material was thoroughly washed in sterile water, surface-disinfected by soaking in 70% ethanol for 30 sec and 0.1% mercuric chloride (HgCl_2_) solution for 2 min, and rinsed in sterile demineralised water. The plant material was subsequently rinsed in sterile demineralised water. Small pieces of inner tissues and needles were placed on aqueous agar (distilled water and 1.5% agar-agar) supplemented with antibiotic streptomycin (3 mg/100 mL) in petri plates and incubated at 28 ± 2°C until fungal growth was initiated. The tips of the fungal hyphae were removed from the aqueous agar and placed on mycological medium, that is, potato dextrose agar (PDA: 300 g/L diced potatoes, 20 g/L dextrose and 20 g/L agar) or the Sabouraud agar (SA: 40 g/L dextrose, 10 g/L peptone, and 20 g/L agar). After several days of incubation, the purity of each fungal culture was assessed by examination of colony morphology. After purifying the isolates several times as described above, the final pure cultures were transferred to PDA slant tubes. As controls, uncut, surface-disinfected, and nondisinfected pieces were also placed on the same agar to check for contaminated fungi.

### 2.3. Identification of Endophytic Fungi

#### 2.3.1. Morphological Examination

The fungi were identified based on morphological characteristics according to the methods described by Kong and Qi [12]. Colony descriptions were based on observations on PDA under ambient daylight conditions. Growth rates at 20, 25, 30, 35, and 40°C were determined after 72 h following published protocols [13, 14]. Microscopic observations and measurements were made from preparations that were mounted in lactic acid. Conidiophore structure and morphology were described from macronematous conidiophores obtained from the edge of conidiogenous pustules or fascicles during the maturation of conidia, which usually occurred after 4–7 days of incubation.

#### 2.3.2. Molecular Examination and Phylogenetic Analyses

For DNA extraction, mycelia were transferred from PDA into 250 mL Erlenmeyer's flasks containing potato-dextrose broth without shaking. After 5 days of growth at 28 ± 2°C, approximately 100 mg of the mycelial biomass was harvested. The genomic DNA was isolated using the Qiagen DNeasy Mini Kit according to the manufacturer's instructions.

 The isolated DNA was diluted in sterile water and stored at 4°C. PCR was performed using the primers ITS4 (5′-TCCTCCGCTTATTGATATGC-3′) and ITS5 (5′-GGAAGTAAAAGTCGTAACAAGG-3′) [15]. The reaction was performed in a 25 *μ*L final volume containing 0.1 *μ*g of genomic DNA, 10 pM of each primer, 1 × *Taq* pol. buffer, 1.5 mM MgCl_2_, 0.2 mM dNTPs, and 1 U of *Taq* DNA polymerase. The following PCR thermal cycle parameters were used: 94°C for 3 min, 35 cycles of 30 s at 94°C, 40 s at 55°C, and 35 s at 72°C and a final extension at 72°C for 7 min.

 The amplified products were examined by electrophoresis in 1.5% agarose gels in TAE buffer, purified using a PCR clean-up kit (A&A Biotechnology), and sequenced using the Applied Biosystems 3730 DNA Analyser (PE Applied Biosystems). As an underlying basis to identify the fungi, the sequences were manually edited and compared with available data from GenBank databases (National Centre for Biotechnology Information website; http://www.ncbi.nlm.nih.gov/) using the BLASTN program [16].

 Fungal rDNA-ITS sequences were submitted to GenBank (accession numbers are listed in Table 1). The sequences that we obtained and those from Genbank (closest identified relatives based on a BLAST search) totalled 36 and were subsequently used for phylogenetic analyses to identify endophytes. The original sequences were edited using Sequencher version 4.0 software (Gene Codes Corp., Ann Arbour, Mich, USA) and aligned using the Clustal W version 1.8 program [17]. The phylogenetic reconstruction was calculated using the neighbour-joining (NJ) algorithm and the maximum-parsimony (MP) method with *Schizosaccharomyces pombe* as an outgroup. NJ analysis was conducted using the MEGA version 4.0 [18] software with bootstrap values calculated from 1000 replicate runs. MP analysis was performed using the PAUP version 4.0 beta 10 program [19] with bootstrap values (1000 replicate runs).

### 2.4. Cultivation of Endophytic Fungi

Each isolated strain of fungi was grown in Sabouraud's broth consisting of 40 g/L dextrose and 10 g/L peptone. Agar blocks containing fungal mycelium were used as the inoculum. The endophyte was grown in a 500 mL Erlenmeyer's flask containing 100 mL of liquid broth (pH 5.6) for a period of 7 days at 28 ± 2°C at 220 rpm in an incubator shaker [20].

### 2.5. Bioactivity Assay

The cultures (mycelia and broths) were, respectively, collected at 24 h intervals for 7 days of fermentation. Mycelia were thoroughly washed with sterile distilled water and homogenised in a cell disintegrator followed by extraction with ethyl acetate. The culture broths were filtered through two layers of cheesecloth, and the filtrates were extracted three times with an equal volume of ethyl acetate. The solvent was blended and concentrated in a vacuum at 35°C. Crude extracts obtained were stored at −20°C until assayed.

The brine shrimp lethality assay, which is an effective and rapid assay to screen potential cytotoxic activity [21], was applied to determine the general toxicity of these endophytic fungi strains from *A. macrosperma*. Brine shrimp (*Artemia salina*) nauplii (the eggs were commercially obtained from Bohai Pharmaceuticals Group, Inc., Yantai, China) hatched after 48 h and tested for LC_50_ values according to Meyer et al. Podophyllotoxin was used as a positive control, and DMSO (1%) was used both as a solvent and negative control. The tests were performed in triplicate and were repeated a total of five times.

The antitumour activity was studied using MTT assays. The following cell lines obtained from the American Type Culture Collection (Manassas, Va, USA) were used: HepG2 cells, MCF7 cells, A549 cells, SGC-7901 cells, and HeLa cells. The tumour cells were maintained in the Roswell Park Memorial Institute 1640 medium (RPMI 1640 medium; Gibco BRL Life Technologies) supplemented with 10% heat-inactivated foetal bovine serum (FBS), 1% glutamine, 100 *μ*g/mL streptomycin, and 2.5 *μ*g/mL amphotericin B (Sigma-Aldrich). The cells were incubated at 37°C in an atmosphere of 5% CO_2_ and 95% air with more than 95% humidity. Cell viability was determined using a colorimetric MTT assay as previously described [22]. All of the tests were performed in triplicate. The optical density (OD) was read using a Benchmark microplate reader at a wavelength of 578 nm (Bio-TEK, USA), and growth inhibition was calculated using the formula listed below. IC_50_ values of the cells were calculated using the NDST software as follows:

(1) $$\text{Inhibition rate}\;\left(\text{IR}\right)\;\%=\left(1-\frac{\text{O}{\text{D}}_{\text{treated well}}}{\text{O}{\text{D}}_{\text{control well}}}\right)\times 100\%.$$

## 3. Results

**Figure 1:**
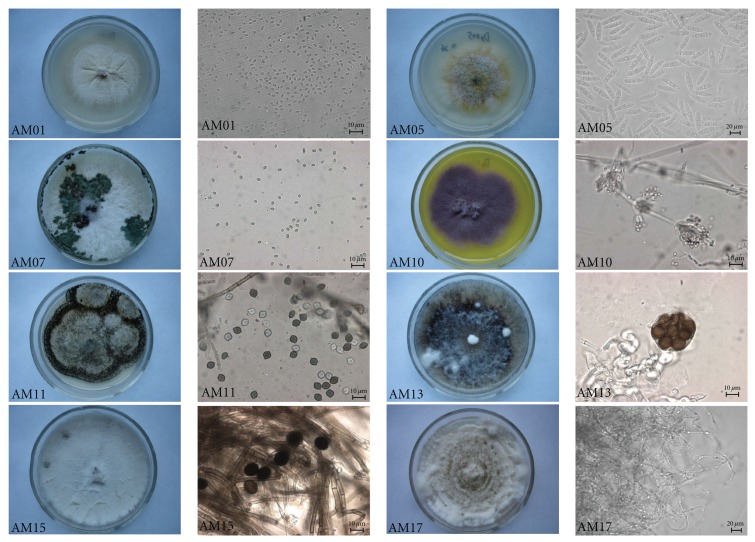
The morphology (colony appearance, conidia, and hypha) of some endophytic fungi isolated from *A. macrosperma. *

According to microscopic characteristics and ITS-rDNA sequences, 17 isolates of fungal endophytes from *A. macrosperma* were obtained. Among the identified fungi, 11 isolates belonged to 5 different taxa (*Acremonium furcatum*, *Cylindrocarpon pauciseptatum*, *Trichoderma citrinoviride*,* Paecilomyces marquandii,* and* Chaetomium globosum*). The other six isolates were grouped into the species of ascomycete *Incertae sedis* with limited morphological and molecular data. Fungal rDNA-ITS sequences obtained in this study were deposited in GenBank (accession numbers: JN596334–JN596350). Table 1 lists the isolates that were obtained from this study along with the best BLAST results. The morphology of some isolates (i.e., characteristics of fruiting structures and spores) is shown in Figure 1.

Fermentation broths of all 17 fungal isolates were tested for cytotoxic activity using the brine shrimp lethality assay. Table 1 shows the diverse LC_50_ values of the isolates, which ranged from 4.86 *μ*g/mL to more than 1000 *μ*g/mL. The LC_50_ value for the positive control podophyllotoxin, which is a well-known cytotoxic lignan, was 2.72 *μ*g/mL. All of the tested materials showed cytotoxic activity except for AM05, AM06, and AM10. Isolates AM07 (4.86 *μ*g/mL), AM11 (7.71 *μ*g/mL), and AM17 (14.88 *μ*g/mL) exhibited significant toxicity against brine shrimp, which were ca. 2 times, 3 times, and 6 times less than podophyllotoxin, respectively.

To assess the effect of fermentation broths on cancer cell proliferation, the MTT assay was used. All of the tested materials inhibited proliferation in a dose-dependent manner. The results of antitumour activity are listed in Table 1. Among the 17 endophytic fungi, 14 (82.4%) showed positive activity (IC_50_ < 100 *μ*g/mL). The percentage of isolates with antitumour activity varied with each cell line. Approximately 76.5% of endophytic fungi cultures displayed antitumour activity in HepG2, MCF7, and SGC-7901 cells, and 82.4% of endophytic fungi cultures displayed antitumour activity in A549 and HeLa cells. In contrast, the isolates showed a lower antitumour effect and relatively higher LC_50_ values in a stomach cancer cell line (SGC-7901) as well as a higher antitumour effect and relatively lower LC_50_ values in a primary non-small cell lung cancer cell line (A549). The isolate AM07 showed the most potent antitumour activity in all of the cell lines examined, which was followed by AM11 and AM17. Thus, the three isolates may have potential as antitumour drugs and require further study. 

## 4. Discussion

**Figure 2:**
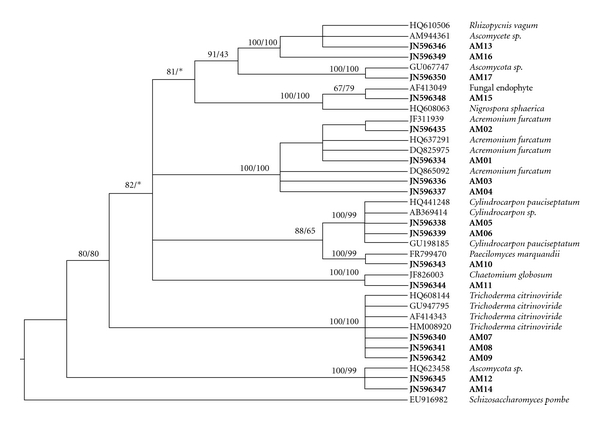
Strict consensus tree reconstructed by maximum parsimony analysis inferred from the nearest neighbours of endophytic fungi isolated from *Actinidia macrosperma*. Bootstrap values exceeding 50% of NJ and MP analysis (MP/NJ) were indicated above branch. Asterisks represent the position of an additional node supported by MP analysis only and collapsed in NJ analysis.

According to classical mycology, most species of endophytic fungi have been described based on their morphological features such as ascospore and peridium morphology, gleba colour, odour, and other organoleptic characteristics [23]. However, these fungi were difficult to identify at the species level. The use of morphological features was problematic for phylogenetic systematics of hypogeous ascomycetes due to a small set of morphological characteristics and homoplasy [23]. In this study, 17 endophytic fungal strains were identified using their microscopic characteristics and confirmed using their ITS-rDNA sequences. The sequences of close relatives were obtained from GenBank to reconstruct the phylogenetic tree. In this tree (Figure 2), all of the 17 endophytes belonged to the phylum Ascomycota, which agreed with the statistical results showing that Basidiomycota endophytes were seldom found within plants [24].

In Figure 2, AM01, AM02, AM03, AM04, and *Acremonium furcatum *were in the same clade with a 100% bootstrap value (MP and NJ analyses). AM07, AM08, AM09, and *Trichoderma citrinoviride* formed a monophyletic clade with a 100% bootstrap value (MP and NJ analyses). AM05, AM06, and *Cylindrocarpon pauciseptatum* remained within an unresolved polytomy with a strong support value. Similarly, *Paecilomyces marquandii *and *Chaetomium globosum *were sister to the isolates AM10 and AM11, respectively (MP/NJ: 100%/99%, 100%/100%, resp.). These results are in agreement with our observations based on their microscopic characteristics. Therefore, these isolates were named according to the phylogenetic tree. The isolates AM12, AM13, AM14, AM15, AM16, and AM17 were not given a genus or species name due to the limitations inherent in DNA-based identification. In the tree, AM13, AM16, AM17, *Ascomycete sp*., *Ascomycota sp*., and *Rhizopycnis vagum* formed a clade with 100% support in MP/NJ analyses. However, the identification of the isolates AM13, AM16, and AM17 at the species or clade level failed. Although phylogenetic relationships indicated a great similarity in ITS sequence between *Rhizopycnis vagum* and AM13, AM16, and AM17, divergent morphology between *Rhizopycnis vagum* and the three isolates suggested that the isolates AM13, AM16, and AM17 should be grouped with ascomycete *Incertae sedis* sp., Finally, AM15 was grouped into a species of ascomycete *Incertae sedis* with limited molecular data. 

Today, more and more studies have focused on the endophytic fungi extracted from various medicinal plants for their antitumour activity. Huang et al. [25] isolated 172 endophytic fungi from three medicinal plants and tested their fermentation broths for cytotoxicity. Their results showed that the percentage of the broths at a dilution at 1 : 50 displayed a cytotoxic activity of 50% growth inhibition. Moreover, Phongpaichit et al. [26] obtained 65 crude extracts from 51 endophytic fungi isolated from *Garcinia *plants and assessed these extracts for various bioactivities. Their results reveal that 40.0%, 12.7%, and 11.1% of fermentation broths display cytotoxicity against Vero, KB, and NCI-H187 cells, respectively. Several studies on endophytic fungi have obtained noteworthy isolates for synthesising bioactive compounds. Some of these compounds have been used for novel drug discovery [27]. However, to the best of our knowledge, there was no relevant study on the isolation of endophytic fungi from the original *A. macrosperma* plant. In China, this medicinal plant has been used for years by those living near their distribution range in tropical and subtropical rainforest areas. Plant medicine is attractive than chemical treatment methods due to its low cost and less detrimental impact on the environment. However, with the overexploitation of the rainforest, *A. macrosperma* is under threat of extinction. As the ability of certain endophytes to biosynthesis certain phytochemicals that were originally associated with this host plant, the study of endophytic fungi from *A. macrosperma* materials is important.

In summary, the results of this study further our microecological understanding of endophytic fungi in their *A. macrosperma* host and demonstrate that some of these fungi possess anticancer potential. The toxicity of endophytic fungi was evaluated *in vitro* using the brine shrimp short-term bioassay, a useful tool to test plant extract bioactivity that correlates with cytotoxic and antitumour properties [28]. Based on the results of the present study, a significant correlation was observed between brine shrimp lethality and cytotoxicity in cancer cell lines. The toxicity of the samples in the brine shrimp model was approximately 2–4 times higher than that shown for the cancer cell lines. 

In this study, *Trichoderma citrinoviride *was the most active fungi. All of the cell lines tested were highly sensitive to the fermentation broth of *T*.* citrinoviride*. Fungi from the genus *Trichoderma *have been extensively studied for their biocontrol potential and are among the most commercially marketed (approximately 60% of all fungal biological control agents) as biopesticides, biofertilisers, and soil amendments [29, 30]. Traditionally, the biocontrol mechanisms that are proposed for *Trichoderma* species act on pathogens and include mycoparasitism, antibiosis, and competition for nutrients and space [31]. Many species produce a wide heterogeneous range of bioactive metabolites that may contribute to their mycoparasitic and antibiotic action [32].

The species composition of endophytic microorganisms may depend on the plant age, genotype, sampled tissue, host type, and season of isolation [2]. During the long coevolution of endophytes and their host plants, many endophytes biosynthesise phytochemicals that are originally associated with the host plant [33]. These have potential for therapeutic purposes and can be used prolifically as research tools. Thus, the isolation and identification of the biologically active substances from fermentation broths of *A. macrosperma* was under way. In the meantime, based on the fact that ITS1 and ITS2 may be too species-specific and the use of, for example, 28 S rRNA or rpb2, may help align the unknown sequences at least to a genus level, further molecular studies of unclear isolates are urgently needed.

## Figures and Tables

**Table 1:** Identification and biological activity of endophytic fungi isolated from *A. macrosperma* ^a^.

GenBank acc. no.	Endophytic fungi code	Identified species^**c**^	% Similarity	Cytotoxic activity LC_50_ (*μ*g/mL)	Antitumor activity LC_50_ (*μ*g/mL)				
					HepG2	MCF7	A549	SGC-7901	HeLa
JN596334	AM01	*Acremonium furcatum *(HQ637291)	100	325.72 (198.00~792.90)	87.75 (45.86~165.40)	88.70 (38.94~133.34)	85.77 (54.82~176.32)	>100	99.08 (25.62~383.20)
JN596335	AM02	*Acremonium furcatum *(JF311939)	100	206.31 (126.20~437.21)	81.05 (56.32~120.06)	76.29 (28.74~165.30)	70.98 (36.26~98.87)	92.56 (53.24~322.14)	71.83 (57.64~89.51)
JN596336	AM03	*Acremonium furcatum *(DQ865092)	99	262.94 (184.72~421.58)	90.63 (65.52~142.30)	88.65 (52.72~128.80)	84.80 (53.73~128.73)	94.07 (79.16~111.80)	83.92 (62.20~131.03)
JN596337	AM04	*Acremonium furcatum *(DQ865092)	100	291.30 (236.63~358.67)	93.60 (85.27~127.54)	91.98 (55.45~167.76)	89.15 (44.08~122.66)	97.74 (63.22~143.24)	94.9 (65.72~157.74)
JN596338	AM05	*Cylindrocarpon pauciseptatum* (HQ441248)	98	>1000	>100	>100	>100	>100	>100
JN596339	AM06	*Cylindrocarpon pauciseptatum* (AB369414)	100	>1000	>100	>100	>100	>100	>100
JN596340	AM07	*Trichoderma citrinoviride *(HQ608144)	100	4.86 (2.95~7.55)	3.13 (2.26~5.72)	3.07 (2.82~6.01)	2.00 (1.89~5.43)	3.44 (2.08~5.63)	2.88 (2.14~6.37)
JN596341	AM08	*Trichoderma citrinoviride *(HQ608144)	99	35.88 (34.02~37.84)	24.63 (12.34~50.77)	22.10 (15.85~31.17)	16.22 (10.45~27.31)	23.66 (20.22~44.38)	22.08 (10.28~53.24)
JN596342	AM09	*Trichoderma citrinoviride *(AF414343)	98	21.20 (11.10~32.20)	11.03 (4.29~19.72)	10.64 (7.70~20.10)	8.83 (4.42~14.09)	12.57 (9.41~18.09)	10.11 (5.82~24.40)
JN596343	AM10	*Paecilomyces marquandii *(FR799470)	100	>1000	>100	>100	>100	>100	>100
JN596344	AM11	*Chaetomium globosum *(JF826003)	99	7.71 (7.30~8.12)	3.87 (2.96~5.45)	3.92 (2.74~7.14)	3.14 (2.27~6.62)	4.05 (3.01~6.29)	3.86 (2.87~7.33)
JN596345	AM12	Ascomycete *Incertae sedis* sp. (HQ623458)	100	124.54 (67.51~183.57)	53.77 (21.10~82.23)	48.63 (23.11~61.76)	46.92 (21.10~76.50)	74.77 (64.83~86.23)	45.47 (18.89~67.71)
JN596346	AM13	Ascomycete *Incertae sedis* sp. (AM944361)	99	228.32 (170.54~311.08)	75.70 (69.05 ~82.99)	75.22 (69.59~81.30)	58.21 (39.79~85.15)	85.39 (35.62~119.04)	69.14 (23.66~108.74)
JN596347	AM14	Ascomycete *Incertae sedis* sp. (HQ623458)	99	179.09 (131.23~243.95)	46.64 (31.10~62.29)	50.33 (42.20~60.02)	47.47 (20.03~59.99)	68.10 (29.74~153.40)	44.43 (30.07~52.90)
JN596348	AM15	Ascomycete *Incertae sedis* sp. (AF413049)	95	490.79 (258.30~682.34)	>100	>100	89.35 (63.32~112.31)	>100	98.26 (55.40~182.31)
JN596349	AM16	Ascomycete *Incertae sedis* sp. (AM944361)	97	61.57 (36.53~103.61)	31.83 (10.98~53.26)	35.88 (34.02~37.84)	28.93 (20.15~33.29)	39.04 (33.19 ~45.92)	33.36 (26.41~42.15)
JN596350	AM17	Ascomycete *Incertae sdis* sp. (GU067747)	100	14.88 (14.05~15.81)	7.90 (5.33~20.15)	8.59 (5.27~16.21)	6.79 (4.63~12.28)	10.04 (6.29~14.95)	8.53 (6.22~19.65)
/	Podophyllo-toxin^b^	/	/	2.72 (2.38~3.50)	0.94 (0.66~2.03)	1.02 (0.75~1.99)	0.75 (0.58~1.77)	1.37 (0.98~2.23)	0.84 (0.62~1.52)

^a^ All determinations were done in triplicate, 95% confidence limits in parentheses. No mortality with negative control group (1% DMSO).

^b^ Positive control group; ^c^ Closest identified relatives were shown in brackets (by BLAST search).
